# Incidence and Predictors of Healthcare-Associated Infections in Patients Admitted to a Temporary Intensive Care Unit during the COVID-19 Pandemic Waves: A Two-Year (2021–2023) Retrospective Cohort Study in Rome, Italy

**DOI:** 10.3390/antibiotics13090842

**Published:** 2024-09-04

**Authors:** Antonio Sciurti, Valentina Baccolini, Mariateresa Ceparano, Claudia Isonne, Giuseppe Migliara, Jessica Iera, Francesco Alessandri, Giancarlo Ceccarelli, Carolina Marzuillo, Guglielmo Tellan, Maria De Giusti, Francesco Pugliese, Paolo Villari, the Collaborating Group

**Affiliations:** 1 Department of Public Health and Infectious Diseases, Sapienza University of Rome, 00185 Rome, Italypaolo.villari@uniroma1.it (P.V.); 2 Department of Life Sciences, Health, and Health Professions, Link Campus University, 00165 Rome, Italy; 3 Management and Health Laboratory, Institute of Management, Department EMbeDS, Sant’Anna School of Advanced Studies, 56127 Pisa, Italy; 4 Department of Anaesthesia and Intensive Care Medicine, Umberto I Teaching Hospital, Sapienza University of Rome, 00185 Rome, Italy; 5 Department of General and Specialist Surgery “P. Stefanini”, Sapienza University of Rome, 00185 Rome, Italy

**Keywords:** healthcare-associated infections, COVID-19, infection prevention and control, *Acinetobacter baumannii*

## Abstract

To manage the number of critical COVID-19 patients, Umberto I Teaching Hospital in Rome established a temporary ICU on March 1, 2021. This study investigated the incidence and risk factors of healthcare-associated infections (HAIs) among these patients during various COVID-19 waves. Patients were grouped by admission date according to the dominant SARS-CoV-2 variant prevalent at the time (Alpha, Delta, Omicron BA.1, Omicron BA.2, Omicron BA.5, and Omicron XBB). First-HAI and mortality rates were calculated per 1000 patient-days. Predictors of first-HAI occurrence were investigated using a multivariable Fine–Gray regression model considering death as a competing event. Among 355 admitted patients, 27.3% experienced at least one HAI, and 49.6% died. Patient characteristics varied over time, with older and more complex cases in the later phases, while HAI and mortality rates were higher in the first year. Pathogens responsible for HAIs varied over time, with first *Acinetobacter baumannii* and then *Klebsiella pneumoniae* being progressively predominant. Multivariable analysis confirmed that, compared to Alpha, admission during the Omicron BA.1, BA.2, BA.5, and XBB periods was associated with lower hazards of HAI. Despite worsening COVID-19 patient conditions, late-phase HAI rates decreased, likely due to evolving pathogen characteristics, improved immunity, but also better clinical management, and adherence to infection prevention practices. Enhanced HAI prevention in emergency situations is crucial.

## 1. Introduction

Italy was one of the first countries to face the acute phase of the SARS-CoV-2 pandemic, which exerted significant pressure on the national healthcare service. The capacity to manage critically ill patients was soon strained, with intensive care units (ICUs) reaching capacity in the early months of 2020 [1,2]. In response to the overwhelming demand and in anticipation of subsequent pandemic waves, hundreds of additional hospital beds were created all over the country, including temporary wards [3]. Similarly to other countries, temporary ICUs were set up to address the shortage of critical care beds, either by adapting existing non-ICU wards in hospitals [4,5,6,7] and non-medical facilities [8,9] or by building new structures from the ground up [10]. Training and reassignment of medical and nursing personnel to operate within these ICUs was also a key strategy [4,5,7,10], leading to enhanced flexibility in the healthcare response and the optimization of resource allocation [11].

Within this emergency context, maintaining optimal infection prevention and control (IPC) practices and preventing the transmission of infectious agents within healthcare settings was a challenge: despite best efforts, the sheer volume of patients overwhelmed these facilities and often exceeded their intended capacities, resulting in high patient density and limited space for isolation, or inadequate areas for putting on and taking off -personal protective equipment [12,13]. In addition, the occurrence of healthcare-associated infections (HAIs) was facilitated in ICU-hospitalized COVID-19 patients, who were found to be particularly vulnerable to superinfections compared to non-COVID-19 patients, as they often required invasive procedures and had prolonged hospital stays [14,15,16,17,18,19]. Furthermore, the frequent use of antibiotics in these patients, especially at the beginning of the pandemic, seems to have increased antimicrobial resistance and the incidence of multidrug-resistant organisms [20], exacerbating the situation further.

Several studies have already investigated the occurrence of HAIs among patients diagnosed with COVID-19 who were admitted to ICUs, confirming higher levels of antimicrobial resistance [21] and a negative impact of HAIs on the survival of these patients [22,23]. However, the impact of the different COVID-19 waves on the occurrence and characteristics of HAIs in ICU patients has not yet been fully explored [24], particularly in specific settings such as temporary ICUs. Therefore, the aims of this retrospective cohort study were to quantify the incidence of HAIs in COVID-19 patients admitted to a temporary ICU at Umberto I Teaching Hospital of Rome over the two-year period following its establishment, and to investigate the factors associated with the occurrence of HAIs. To the best of our knowledge, this is the first study investigating the occurrence of HAIs in this setting during the COVID-19 pandemic.

## 2. Materials and Methods

### 2.1. Patients and Data Collection

We retrospectively analysed patients admitted to a temporary ICU of the Umberto I Teaching Hospital of Rome from the 1st March 2021 (i.e., the day it opened) to the 30th of June 2023 (i.e., the day the last patient was admitted to the ICU). The follow-up was extended until the last patient was discharged from the ICU (i.e., 15th July 2023, the day it closed). This temporary ICU was set up in a prefabricated facility, connected by a tunnel to the hospital. Data on ICU patients and HAIs were retrieved from the active HAI surveillance system that was conducted on the ward. The detailed methodology of such a surveillance system is described elsewhere [25]. In short, diagnostic criteria for detecting HAIs were derived from the National Healthcare Safety Network of the Center for Disease Control [26] and the European Centre for Disease Prevention and Control [27] protocols. All patients hospitalized to the ICU for a minimum of two consecutive days underwent monitoring until their discharge from the ICU. The onset of blood infections related to central lines (catheter-related bloodstream infections, CRBSIs), pneumonias associated with mechanical ventilation (ventilation-associated pneumonias, VAPs), and urinary tract infections associated with bladder catheters (catheter-associated urinary tract infections, CAUTIs) occurring from 48 h after insertion of the device was registered. Additionally, the surveillance system recorded data on the occurrence of bloodstream infections of unknown origin (BUOs) and surgical site infections (SSIs) arising either 48 h after ICU admission or within 30 days post-surgery, respectively. BUOs are specifically defined as laboratory-confirmed bloodstream infections not resulting from an infection originating at another body site [27], while SSIs are infections occurring in proximity to or at the incision site and/or deeper tissue spaces [26]. 

Patient information was systematically gathered through a structured form comprising four sections: (1) patient demographics and hospitalization details (including ICU admission date, admission type, discharge date, discharge status, coexisting conditions, simplified acute physiology score II [SAPS II], and presence of COVID-19 pneumonia); (2) exposure to invasive devices, i.e., start and end date of urinary catheterization, central venous catheterization, and mechanical ventilation, including whether these devices were in use within 48 h prior to the onset of infection; (3) antibiotic treatment, including the drug administered and the start and end dates of treatment for each drug; (4) diagnosed HAIs and related microbiological cultures, including the infection site, onset date, and microbiological confirmation details (sample collection date and identified microorganisms). The antimicrobial susceptibility profiles of microorganisms were determined based on the classification proposed by Magiorakos et al, where applicable [28]. Laboratory confirmation of SARS-CoV-2 infection is defined as a positive result of a real-time reverse transcription polymerase chain reaction assay of nasal and pharyngeal swabs [29]. 

Patients were classified according to ICU admission date into the corresponding period of dominance of SARS-CoV-2 variants in Italy. Cut-off dates between periods of SARS-CoV-2 variant dominance were conventionally defined based on when one variant was reported to have become dominant over the others, according to the reports provided by the Italian National Institute of Health [30], as follows: (1) Alpha period (from 28 December 2020 to 18 June 2021); (2) Delta period (from 19 June 2021 to 31 December 2021); (3) Omicron BA.1 period (from 1 January 2022 to 12 March 2022); (4) Omicron BA.2 period (from 13 March 2022 to 6 June 2022); (5) Omicron BA.5 period (from 7 June 2022 to 12 February 2023); and (6) Omicron XBB period (from 13 February 2023 to 15 July 2023).

The institutional ethics committee of the Umberto I Teaching Hospital of Rome reviewed and approved this study (reference number 0282/2024).

The study was reported according to the STROBE checklist for cohort studies [31] (Table S1).

### 2.2. Statistical Analysis

Descriptive statistics by period of SARS-CoV-2 variant dominance were obtained using mean and standard deviation (SD) for continuous variables and proportions for dichotomous and categorical variables, while their association with the variant periods was tested using the Kruskal–Wallis and Pearson’s chi-squared tests, as appropriate. The first-HAI and mortality rates, with their 95% confidence intervals (95% CIs), were estimated per 1000 patient-days by period of SARS-CoV-2 variant dominance assuming a Poisson distribution. For this study, we defined the use of invasive devices (i.e., urinary catheterization, central venous catheterization, and mechanical ventilation) as having used any device for at least two consecutive days. Similarly, antibiotic consumption was coded as having used any antibiotic agent for at least two consecutive days in a systemic administration (i.e., enteral or parenteral) for a different clinical reason than the first HAI in the time period from ICU admission to the day before HAI onset or to the date of discharge.

Considering the event of death as a competitive event, a competing-risk regression model was used to explore the effect of the SARS-CoV-2 variant dominance period on first-HAI occurrence. Specifically, a multivariable Fine–Gray regression model for proportional hazards was used, providing estimates of the sub-distribution hazard ratio (SHR) (i.e., the relative change in the instantaneous rate of the occurrence of the event in those subjects who were event-free or who had experienced the competitive event) and its associated 95% CI [32,33]. The main exposure of interest (i.e., the period of SARS-CoV-2 variant dominance) was adjusted for the potential confounders of the association, based on expert knowledge [34]. Only the most frequent antibiotic exposures (i.e., carbapenems, glycopeptides, penicillins) were included in the model. Missing SAPS II values (35.5%) were imputed using univariate multiple imputation, applying a truncated multivariable linear regression model that constrained imputations between 0 and 163, the minimum and maximum possible SAPS II scores. Along with the outcome, all variables from the multivariable survival analysis were incorporated into the imputation models. To address the high rate of missing data, 20 imputed datasets were generated. [35]. As a result, the final models included the following variables: period of SARS-CoV-2 variant dominance (Alpha, Delta, Omicron BA.1, Omicron BA.2, Omicron BA.5, and Omicron XBB periods); age (continuous, years); gender (female or male); SAPS II (continuous); coexisting conditions (no coexisting condition, one coexisting condition, two coexisting conditions or more); being intubated at admission (non-intubated, intubated with COVID-19 pneumonia, intubated for other reasons); use of carbapenems before first HAI (continuous, days); use of glycopeptides before first HAI (continuous, days); and use of penicillins before first HAI (continuous, days). Interaction terms between the variables were tested with statistical significance set at <0.05. The proportionality assumption was checked by testing the statistical significance of interaction terms involving failure time, one term at a time.

All analyses were performed using STATA (StataCorp LLC, 4905 Lakeway Drive, College Station, TX, USA), version 17.0. A two-sided *p*-value < 0.05 was considered statistically significant.

## 3. Results

### 3.1. Characteristics of Patients

**Figure 1:**
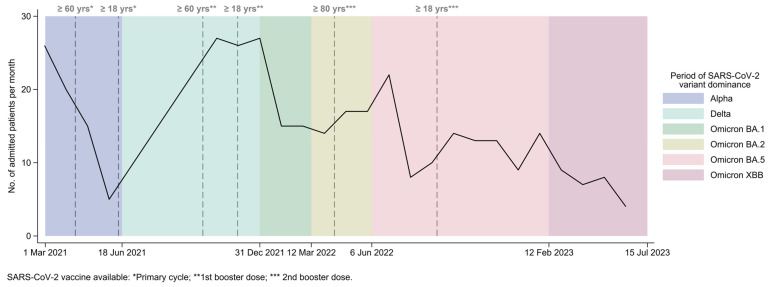
ICU-admitted patients per month (N = 355) by period of SARS-CoV-2 variant dominance. The dashed vertical lines denote the date when the SARS-CoV-2 vaccine, both primary cycle and booster doses, became available to the population, based on age groups in the Lazio Region.

From the 1st March 2021 to 30th June 2023, 355 patients were hospitalized in the temporary ICU of the Umberto I Teaching Hospital of Rome. Admissions decreased over time, with peaks in March 2021, November 2021, December 2021, and January 2022 (Figure 1).

Accordingly, the number of admitted patients decreased from the Alpha (N = 66) to the Omicron XBB dominance periods (N = 38), with the exception of Omicron BA.5 (N = 107). Cumulative patient-days from admission ranged from 658 (Omicron BA.2) to 1765 (Omicron BA.5) (Table 1). 

Males were consistently predominant (54.5–75.6%, *p* = 0.258), but the characteristics of hospitalized patients varied significantly across study periods, examples being mean age (increasing from 60.3 ± 12.9 years in the first period to 72.5 ± 14.6 years in the last period, *p* < 0.001), source of admission (emergency department varied from 71.1% during Omicron BA.1 to 35.5% during BA.5, *p* < 0.001), and number of coexisting conditions (patients with two or more conditions increased from 19.7% during Alpha to 52.6% during Omicron XBB). As for the type of coexisting condition, hypertension was the most prevalent across the study periods, increasing significantly from 30.3% in the Alpha period to 57.9% in the Omicron XBB months, followed by diabetes, whose prevalence was stable (*p* = 0.235). Approximately one quarter of the patients had a chronic heart condition during Omicron XBB or an active neoplasm during Omicron BA.2 and BA.5. 

Among those with an available SAPS II score (229 patients), the lowest value was observed in patients admitted during the Alpha period (28.7) compared to those admitted during other periods (ranging from 37.2 to 59.0). On the one hand, the proportion of non-intubated patients at admission was 83.3% during Alpha and then decreased over time, reaching the lowest value in the Omicron BA.5 months (51.4%), when approximately 30% of patients were intubated without COVID-19 pneumonia. On the other hand, the proportion of patients intubated with COVID-19 pneumonia varied over time, reaching the highest values in the second and third periods (39.6% and 28.9%, respectively) and decreasing subsequently (10.9%, 18.7% and 21.1% in the Omicron BA.2, Omicron BA.5 and Omicron XBB months, respectively). Regarding invasive devices, their use was comparable throughout the study; e.g., the proportion of patients with a central venous catheter ranged from 65.2% to 81.3% (*p* = 0.404), while those with a urinary catheter represented over 95% in every period (*p* = 0.294). By contrast, the use of mechanical ventilation was at a minimum during the Alpha period (50.0%), while the following periods showed the proportion of patients who were ventilated ranging from 52.6% to 77.4% (*p* = 0.017). 

Antibiotic consumption was variable across the study periods. Penicillins were the most administered class (201 patients, 59.1%), ranging from an average of 7.6 days in the Alpha period to 12.5 days in the Omicron XBB period. Glycopeptide consumption (186 patients, 52.4%) ranged from 10.1 average days in the Delta period to 16.1 in the Omicron XBB period. Antifungal agents (176 patients, 49.6%) were used for longer, i.e., between around 13 and 15 days on average, across the study periods. Carbapenems (172 patients, 48.5%) were taken on average for between 12.5 and 11.1 days in the Alpha, Delta and Omicron BA.1 periods and between 10.6 and 10.9 days in the Omicron BA.2, Omicron BA.5 and Omicron XBB periods. Extended-spectrum cephalosporins (156 patients, 44.0%) were used for the longest time in the Omicron BA.1 period (13.2 average days) and the shortest in the Omicron BA.2 period (7.3 average days). Macrolides (115 patients, 32.4%), on the other hand, were taken for the shortest time in the Omicron BA.5 period (5.8 average days) and the longest in the Omicron XBB period (9.4 average days) (*p* = 0.006). Polymyxins were the least-used antimicrobial class (93 patients, 26.2%), being taken for 8.9 days on average in the Delta period and up to 14.3 days in the Omicron BA.1 period.

The length of stay in the ICU was similar over time, from 14.8 to 19.1 days on average (*p* = 0.604), but the Delta and Omicron BA.1 months showed the highest proportion of deaths (66.0% and 71.1% of patients, respectively), compared to the other periods (*p* < 0.001). Accordingly, a higher mortality rate per 1000 patient-days was found in the Delta (44.4, 95% CI: 29.7–59.1), and Omicron BA.1 (38.8, 95% CI: 25.3–52.2) periods, compared to the Alpha (20.4, 95% CI: 11.5–29.4), Omicron BA.2 (33.4, 95% CI: 19.5–47.4), Omicron BA.5 (30.6, 95% CI: 22.4–38.8), and Omicron XBB (17.9, 95% CI: 8.2–27.6) periods, with rates decreasing overall from the Delta to the Omicron XBB period.

### 3.2. Occurrence and Characteristics of HAIs and Isolated Microorganisms

Overall, 138 HAIs occurred during the study period, affecting 27.3% of the patients (Table 2).

The most common HAIs were CAUTIs (52.9%) during Alpha and VAPs in all other periods (from 50.0% to 61.9%), with the only exception being the Omicron BA.2 period, during which VAP and BUO were equally diagnosed (36.4% each). The incidence of patients with at least one HAI was highest during Alpha, Delta, and Omicron BA.1 variants (31.8%, 41.5% and 37.8%, respectively), while the proportion of patients who suffered from two or more superinfections reached a maximum during the first months of the study (15.2% in the Alpha and 15.1% in the Delta period) (*p* = 0.023).

**Figure 2:**
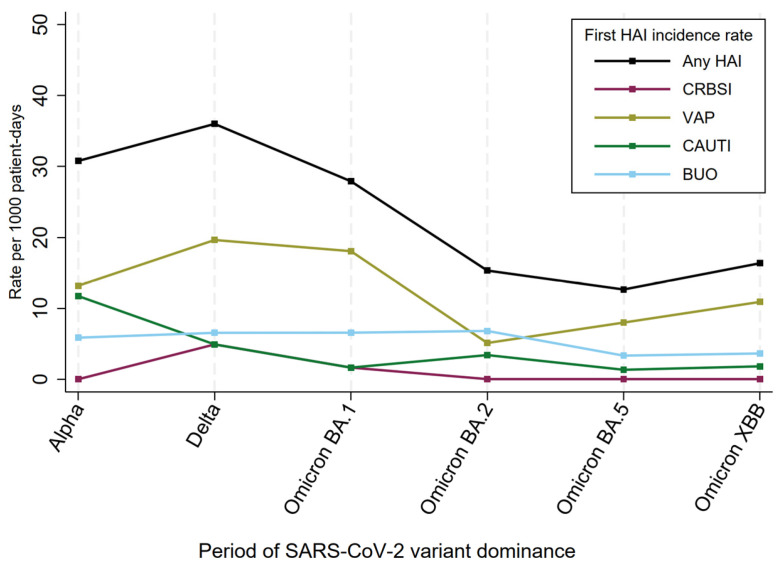
First HAI incidence rates, by period of SARS-CoV-2 variant dominance. BUO: bloodstream infections of unknown origin; CAUTI: catheter-associated urinary tract infection; CRBSI: catheter-related bloodstream infection; HAI: healthcare-associated infection; SARS-CoV-2: severe acute respiratory syndrome coronavirus 2; VAP: ventilation-associated pneumonia.

The time-to-first HAI was similar throughout the study (from 10.3 to 14.5 days), but higher rates per 1000 patient-days were observed in the Alpha (30.8, 95% CI: 17.6–44.0), Delta (36.0, 95% CI: 21.0–51.1), and Omicron BA.1 periods (27.9, 95% CI: 14.6–41.2) compared to the Omicron BA.2 (15.3, 95% CI: 5.3–25.3), Omicron BA.5 (12.7, 95% CI: 7.0–18.3), and Omicron XBB months (16.4, 95% CI: 5.7–27.1) (Figure 2). 

As for the different types of HAI, VAPs showed a trend comparable to the overall incidence rate, peaking during Delta and Omicron BA.1 predominance, and showing the highest incidence rates in all periods except Omicron BA.2, during which BUOs were slightly more represented. The other HAI types recorded some fluctuations over time, but did not exceed the rate of 10 per 1000 patient-days in any period apart from CAUTI during the Alpha variant (11.7, 95% CI: 3.6–19.9). 

**Figure 3:**
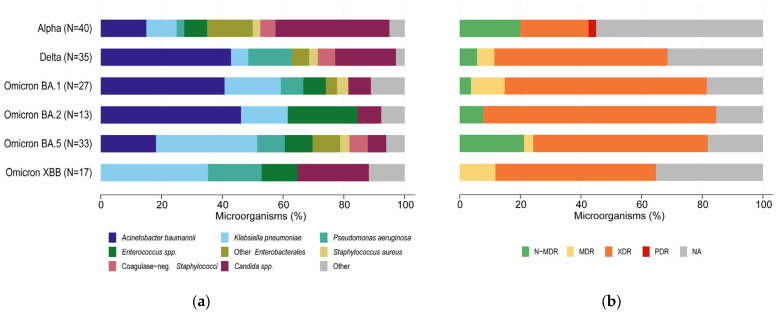
Microorganism (N = 165) (**a**) type and (**b**) antimicrobial susceptibility profile, by period of SARS-CoV-2 variant dominance. SARS-CoV-2: severe acute respiratory syndrome coronavirus 2; N-MDR: non-multidrug-resistant; MDR: multidrug-resistant; XDR: extensively drug-resistant; PDR: pandrug-resistant; NA: not applicable.

Lastly, of the isolated microorganisms (N = 165), *A. baumannii* (N = 44) was the most frequently responsible for HAIs, followed by *Candida* spp. (N = 31) and *K. pneumoniae* (N = 30). *Candida* spp. were prevalent in the Alpha period (37.5%), while *A. baumannii* became progressively predominant subsequently (42.9%, 40.7% and 46.2% in the Delta, Omicron BA.1 and Omicron BA.2 periods, respectively) and *K. pneumoniae* was the most frequently recorded in the last months of the study (33.3% and 35.3% in the Omicron BA.5 and XBB periods, respectively) (Figure 3a). As for the type of HAI, *Candida* spp. was the most frequently isolated agent among CRBSIs (N = 2, 33.3%). For VAPs, *A. baumannii* was the most common microorganism (N = 31, 37.8%), followed by *K. pneumoniae* (N = 20, 24.4%) and *P. aeruginosa* (N = 12, 14.6%). In CAUTIs, *Candida* spp. was the most prevalent pathogen (N = 20, 54.1%), followed by *K. pneumoniae* (N = 6, 16.2%) and *A. baumannii* (N = 4, 10.8%). Finally, among BUOs, *Enterococcus* spp. was the most frequent microorganism (N = 10, 25.0%), followed by *Candida* spp. (N = 9, 22.5%) and *A. baumannii* (N = 8, 20.0%). Regarding the microorganisms’ antimicrobial susceptibility profiles (Figure 3b), only one pandrug-resistant (PDR) isolate was found during the Alpha period (*K. pneumoniae*). However, the proportion of extensively drug-resistant (XDR) isolates tended to increase from the Alpha period (22.5%) to the Omicron BA.2 period (76.9%) and then stabilized during the Omicron BA.5 and XBB periods (57.6% and 52.9%, respectively). The proportion of multidrug-resistant microorganisms (MDRs) was more fluctuating across the study periods and was around 11% in the Omicron BA.1 and Omicron XBB period. Non-multidrug-resistant microorganisms (N-MDR) did not exceed 21% in any period, with no N-MDR isolates found during the Omicron XBB period.

### 3.3. Multivariable Analysis of Occurrence of First HAI

Compared to the Alpha period (Table 3), admission during the various Omicron waves was associated with lower hazards of HAI (Omicron BA.1 SHR: 0.40, 95% CI: 0.16–0.96; Omicron BA.2 SHR: 0.30, 95% CI: 0.13–0.72; Omicron BA.5 SHR: 0.23, 95% CI: 0.11–0.50; and Omicron XBB SHR: 0.34, 95% CI: 0.14–0.82), but this was not the case during the Delta period (SHR: 0.65, 95% CI: 0.31–1.34). 

Male patients showed higher hazards of HAI (SHR: 4.01, 95% CI: 1.68–9.57), but this association decreased over time (SHR: 0.92, 95% CI: 0.88–0.97). Being intubated with COVID-19 pneumonia at admission was associated with greater hazards of HAI (SHR: 1.82, 95% CI: 1.08–3.07), whereas being intubated for other reasons at admission was not associated with the outcome (SHR: 1.54, 95% CI: 0.79–3.01). Longer use of carbapenems was associated with lower hazard of HAI (SHR: 0.87, 95% CI: 0.82–0.93), although this was found to increase over time (SHR: 1.00, 95% CI: 1.00–1.01). Lastly, age, SAPS II, coexisting conditions, and daily use of glycopeptides and penicillins did not show any relationship with the outcome.

## 4. Discussion

In this study, we focused on a temporary ICU ward, specifically built for critical COVID-19 patients, to provide insights into the occurrence of HAIs throughout the pandemic waves. We found that hospitalized patients differed over time in their clinical characteristics. Thus, during the early stages of the pandemic, younger patients with fewer comorbidities were admitted, while in the later phases patients were older and had more coexisting conditions. This shift may be related to the reduced virulence of the Omicron variants [36], and the progressive increase in population immunity due to the increase in numbers of vaccinated individuals [37]. Similarly to other countries, older individuals, as the most at-risk group in the general population, were the first to be targeted by the anti-COVID-19 vaccination campaign in Italy [38,39], and this may have conferred greater protection against SARS-CoV-2 infection and ICU admission in this age group during the early stages of the pandemic. Additionally, the increase in natural immunity following SARS-CoV-2 infections may have also played a role in preventing severe disease and hospitalization in younger people in the later stages of the pandemic [40,41,42]. Nevertheless, despite better baseline patient conditions at the beginning of the pandemic, ICU mortality was higher in these months, peaking during the Delta variant, which was deemed more pathogenic than both the Alpha [43] and Omicron lineages [44,45]. Reasons for this finding are doubtless multifactorial and comparable to those mentioned for ICU hospitalizations, including the decreasing virulence of the variants, with Omicron lineages being progressively less lethal [36,37,46,47], and the increase in patient immunization status [48], with people largely receiving booster doses during the Omicron waves in Europe [49,50]. Interestingly, it should be noted that in our study SAPS II seemed to be unrelated to the mortality and HAI rates. Other studies have confirmed the unreliability of SAPS II in predicting superinfections [51], although SAPS II was able to predict the mortality of ICU-hospitalized COVID-19 patients in a few cases [52,53]. A potential explanation for this result could be the young age of the subjects hospitalized in the early periods and their relative lack of co-morbidities, which may have masked the severity of their condition in our study. However, given the limited availability of such data in our cohort, further studies are needed to definitely establish the correlation between SAPS II and mortality in COVID-19 patients. 

We also observed a higher HAI incidence in the first phases of the pandemic, followed by a substantial decrease in the following year, indicating an association between the admission period and the development of HAIs, as confirmed by the multivariable analysis. In this regard, evidence on the direct effect of SARS-CoV-2 variants on the development of HAIs is limited, and there is no clear conclusion [54], but the progressive improvement in management of critically ill COVID-19 patients, and the availability of new therapeutic options [55], must go some way towards explaining the decrease in ICU mortality and HAI incidence observed in the last year of this study. In addition, it is also possible that ICU healthcare workers improved their adherence to IPC measures, particularly given that the first pandemic waves were characterized by fear of SARS-CoV-2 contagion, higher workload and shortages of personal protective equipment (PPE) [56,57]. In this regard, the availability of PPE has been found to be a relevant factor for IPC practice compliance among healthcare workers [58]. Within this context, the inverse relationship between baseline patient characteristics and HAI incidence could be at least partially explained by the general decrease in intubated patients with COVID-19 pneumonia as the study progressed; in fact, this was identified as a risk factor for HAI in the multivariable analysis. It is well-known that COVID-19 pneumonia is a risk factor for VAPs, due to the respiratory compromise that favours bacterial superinfections [59,60,61,62], and in our study VAPs were more prevalent in the first three periods compared to the subsequent phases. On the other hand, it is also worth mentioning that the clinical and radiological characteristics of SARS-CoV-2 and bacterial pneumonia usually overlap [63], complicating the diagnosis of VAP among these patients, a factor that may have impacted our findings, especially in the first waves. 

As for the other predictors of HAI occurrence, neither patient age, coexisting conditions nor SAPS II were associated with HAI onset, while among the antimicrobial agents, only carbapenems had an impact, showing a preventive effect that increased over time. In this regard, it is noteworthy that across the pandemic phases we observed a change in the resistance profile of microorganisms, which became more resistant to multiple antimicrobial agents, including carbapenems. However, caution is needed in interpreting these findings, as studying the role of antibiotics in the occurrence of HAIs is complex, and antibiotic use is widely acknowledged as a primary driver of antimicrobial resistance [63,64]. For this reason, and given that the pandemic situation has led to the inappropriate prescription of antimicrobial agents by healthcare professionals in different settings [65,66], it will be crucial to implement antimicrobial stewardship programs in healthcare settings to optimize antibiotic consumption and mitigate the emergence and spread of multidrug-resistant bacteria [67]. Lastly, we also found that male gender was positively associated with the outcome, with a tendency to decrease over time, in line with other reports showing a higher prevalence of HAIs in male patients in the European WHO region [68]. However, this association might be the result of an *A. baumannii* outbreak that occurred in the ICU between December 2021 and January 2022 exclusively among male patients, possibly as a consequence of the ICU bed layout, in which patients of the same sex were grouped together. Therefore, this finding highlights the urgent need to implement enhanced IPC measures, an aspect that could limit the spread of microorganisms and prevent future outbreaks. In this regard, it may not be a coincidence that pathogens responsible for HAIs varied markedly over time: while *Candida* spp. were the most common microorganisms in the first period, possibly due to the extensive use of corticosteroids in the treatment of severe COVID-19 pneumonia [69,70] at this time, as recommended by international guidelines [71], *A. baumannii* and *K. pneumoniae* became the predominant pathogens in the subsequent periods, findings that suggest microorganism transmission between patients. Indeed, as shown in other studies conducted in the main ICU ward in the same hospital [14,15] and in line with national and international reports [72,73,74], *A. baumannii* and *K. pneumoniae* were both found to be among the pathogens most commonly responsible for HAIs [75,76], and their spread has been frequently linked to inadequate adherence to hygiene precautions [77].

This study has some strengths and limitations. Since the data derive from an ongoing surveillance program routinely conducted by the Department of Public Health and Infectious Diseases, the main strength is the ability to investigate critical COVID-19 patients and to compare their characteristics and outcomes over a two-year period during different pandemic waves. Moreover, since the staff cohort working in the temporary ICU was relatively stable, it ensured consistency of care over time. Nevertheless, some limitations must be acknowledged. Since the data were gathered using a predefined data collection system, it was not possible to genotype the SARS-CoV-2 variants responsible for the infection in hospitalized patients, nor to determine the patients’ vaccination status or to assess whether their vaccination status aligned with the vaccine rollout by age group. In addition, as per the data collection system, we were not able to assess the different standards-of-care adopted during the pandemic, nor the post-ICU discharge status of patients, even though only the most stable were chosen for discharge. Thirdly, to adjust for clinical severity, we used multiple imputation to address the missing SAPS II values, generating 20 imputed datasets to compensate for the high rate of missing data, but some residual confounding could still be present. Fourth, since this temporary ICU was specifically established for COVID-19 patients, there are no pre-pandemic data available for comparison in such wards. Finally, we did not assess the effect of HAIs on patient mortality, as it was outside the scope of our research. Further studies should be conducted to address this issue.

## 5. Conclusions

This study found that, despite a worsening in the clinical characteristics of COVID-19 patients across the pandemic waves, the occurrence of HAIs was higher during the first phases. Such an improvement in clinical outcomes may be attributed to a combination of multiple factors, including reduced SARS-CoV-2 pathogenicity, increased population immunity and a better understanding of the pathophysiology of COVID-19 and the tools to contain its effects, as well as a better application of IPC practices. Moreover, we observed that a temporary ICU setting can be subject to particular pressure in terms of HAIs. This was evident during the COVID-19 pandemic, but temporary ICU facilities may also be required in future emergency scenarios, where a large number of patients are expected to need critical care, such as during other pandemics, epidemics, natural disasters, or humanitarian crises in resource-limited settings. Therefore, further efforts must be undertaken to strengthen and consolidate HAI prevention and control practices during emergency situations.

## Figures and Tables

**Table 1:** Patient (N = 355) characteristics by period of SARS-CoV-2 variant dominance.

	Period of SARS-CoV-2 Variant Dominance						
	Alpha	Delta	Omicron BA.1	Omicron BA.2	Omicron BA.5	Omicron XBB	*p*-Value
	1 Mar 2021– 18 Jun 2021	19 Jun 2021–31 Dec 2021	1 Jan 2022– 12 Mar 2022	13 Mar 2022– 6 Jun 2022	7 Jun 2022– 12 Feb 2023	13 Feb 2023–15 Jul 2023	
Patients, N	66	53	45	46	107	38	
Cumulative patient-days from admission	978	789	825	658	1765	726	
Age, mean (SD)	60.3 (12.9)	64.4 (11.0)	64.8 (11.5)	68.7 (16.3)	69.5 (13.3)	72.5 (14.6)	<0.001
Age, median (IQR)	61.1 (49.5–70.8)	64.5 (55.2–72.3)	66.0 (59.1–73.6)	72.7 (60.7–82.5)	72.1 (61.6–78.9)	75.1 (64.4–84.1)	
Gender, N (%)							
Female	25 (37.9)	18 (34.0)	11 (24.4)	15 (32.6)	31 (29.0)	18 (47.4)	0.258
Male	41 (62.1)	35 (66.0)	34 (75.6)	31 (67.4)	76 (71.0)	20 (52.6)	
Type of admission to ICU, N (%)							<0.001
Other hospital	2 (3.0)	6 (11.3)	7 (15.6)	9 (19.6)	35 (32.7)	12 (31.6)	
Other ward	19 (28.8)	17 (32.1)	2 (4.4)	10 (21.7)	26 (24.3)	5 (13.2)	
Emergency department	39 (59.1)	29 (54.7)	32 (71.1)	21 (45.7)	38 (35.5)	20 (52.6)	
Other	6 (9.1)	1 (1.9)	4 (8.9)	6 (13.0)	8 (7.5)	1 (2.6)	
Coexisting conditions, N (%)							<0.001
No condition	36 (54.5)	24 (45.3)	18 (40.0)	6 (13.0)	24 (22.4)	7 (18.4)	
One condition	17 (25.8)	13 (24.5)	11 (24.4)	18 (39.1)	36 (33.6)	11 (28.9)	
Two conditions or more	13 (19.7)	16 (30.2)	16 (35.6)	22 (47.8)	47 (43.9)	20 (52.6)	
Coexisting conditions, N (%)							
Hypertension	20 (30.3)	24 (45.3)	20 (44.4)	26 (56.5)	55 (51.4)	22 (57.9)	0.036
Diabetes mellitus	9 (13.6)	7 (13.2)	9 (20.0)	10 (21.7)	27 (25.2)	11 (28.9)	0.235
Obesity	5 (7.6)	8 (15.1)	5 (11.1)	5 (10.9)	4 (3.7)	2 (5.3)	0.18
COPD	2 (3.0)	1 (1.9)	5 (11.1)	11 (23.9)	12 (11.2)	6 (15.8)	0.003
Asthma	1 (1.5)	2 (3.8)	2 (4.4)	0 (0.0)	1 (0.9)	0 (0.0)	0.385
Bronchiectasis	0 (0.0)	0 (0.0)	0 (0.0)	1 (2.2)	0 (0.0)	0 (0.0)	0.241
Pulmonary fibrosis	0 (0.0)	0 (0.0)	0 (0.0)	0 (0.0)	1 (0.9)	0 (0.0)	0.803
Chronic heart disease	3 (4.5)	2 (3.8)	3 (6.7)	2 (4.3)	5 (4.7)	10 (26.3)	<0.001
Chronic kidney disease	1 (1.5)	3 (5.7)	5 (11.1)	10 (21.7)	22 (20.6)	2 (5.3)	<0.001
Chronic liver disease	0 (0.0)	0 (0.0)	0 (0.0)	1 (2.2)	0 (0.0)	0 (0.0)	0.241
Active neoplasm	5 (7.6)	4 (7.5)	4 (8.9)	12 (26.1)	28 (26.2)	4 (10.5)	0.001
Neutropenia	0 (0.0)	0 (0.0)	0 (0.0)	1 (2.2)	0 (0.0)	0 (0.0)	0.241
Organ transplantation	0 (0.0)	1 (1.9)	0 (0.0)	0 (0.0)	1 (0.9)	0 (0.0)	0.696
SAPS II, mean (SD) (N = 229)	28.7 (11.2)	38.7 (9.4)	59.0 (-)	40.8 (17.3)	41.7 (16.3)	37.2 (15.7)	NA
Intubated at admission, N (%)							<0.001
Non-intubated	55 (83.3)	30 (56.6)	26 (57.8)	29 (63.0)	55 (51.4)	24 (63.2)	
Intubated, other reasons	2 (3.0)	2 (3.8)	6 (13.3)	12 (26.1)	32 (29.9)	6 (15.8)	
Intubated with COVID-19 pneumonia	9 (13.6)	21 (39.6)	13 (28.9)	5 (10.9)	20 (18.7)	8 (21.1)	
Use of devices before first HAI, N (%)							
Central venous catheter	49 (74.2)	42 (79.2)	35 (77.8)	30 (65.2)	87 (81.3)	29 (76.3)	0.404
Mechanical ventilation	33 (50.0)	41 (77.4)	33 (73.3)	29 (63.0)	71 (66.4)	20 (52.6)	0.017
Urinary catheter	65 (98.5)	53 (100.0)	43 (95.6)	46 (100.0)	106 (99.1)	38 (100.0)	0.294
Antibiotic consumption in days, mean, SD							
Antifungal agents (N = 176)	14.5 (11.5)	12.7 (8.9)	15.2 (14.8)	12.7 (10.7)	15.8 (13.0)	15.3 (17.7)	0.816
Carbapenems (N = 172)	12.5 (9.2)	11.1 (7.3)	11.1 (6.8)	10.8 (8.2)	10.6 (6.9)	10.9 (8.9)	0.976
Extended-spectrum cephalosporins (N = 156)	8.4 (9.7)	7.8 (6.6)	13.2 (11.8)	7.3 (5.2)	11.6 (10.0)	9.2 (7.8)	0.454
Glycopeptides (N = 186)	10.9 (7.8)	10.1 (6.6)	13.4 (9.5)	10.2 (6.4)	13.2 (10.0)	16.1 (14.6)	0.618
Macrolides (N = 115)	6.0 (4.3)	8.1 (4.9)	8.5 (5.6)	8.1 (2.7)	5.8 (3.0)	9.4 (4.5)	0.006
Penicillins (N = 210)	7.6 (4.3)	8.6 (4.6)	11.3 (8.8)	8.5 (7.8)	9.3 (6.7)	12.5 (8.6)	0.361
Polymyxins (N = 93)	11.8 (6.7)	8.9 (3.9)	14.3 (12.4)	9.4 (9.7)	9.6 (5.1)	11.5 (4.9)	0.638
Length of ICU stay in days, mean (SD)	14.8 (12.2)	14.9 (8.5)	18.3 (15.8)	14.3 (10.7)	16.5 (12.7)	19.1 (18.2)	0.604
ICU deaths, N (%)	20 (30.3)	35 (66.0)	32 (71.1)	22 (47.8)	54 (50.5)	13 (34.2)	<0.001
Mortality rate per 1000 patient-days (95% CI)	20.4 (11.5–29.4)	44.4 (29.7–59.1)	38.8 (25.3–52.2)	33.4 (19.5–47.4)	30.6 (22.4–38.8)	17.9 (8.2–27.6)	NA

CI: confidence interval; COPD: chronic obstructive pulmonary disease; HAI: healthcare-associated infection; ICU: intensive care unit; NA: not applicable; SAPS II: simplified acute physiology score II; SARS-CoV-2: severe acute respiratory syndrome coronavirus 2; SD: standard deviation.

**Table 2:** Occurrence and characteristics of healthcare-associated infections (HAIs) by period of SARS-CoV-2 variant dominance.

	Period of SARS-CoV-2 Variant Dominance						
	Alpha	Delta	Omicron BA.1	Omicron BA.2	Omicron BA.5	Omicron XBB	*p*-Value
	1 Mar 2021–18 Jun 2021	19 Jun 2021–31 Dec 2021	1 Jan 2022– 12 Mar 2022	13 Mar 2022–6 Jun 2022	7 Jun 2022– 12 Feb 2023	13 Feb 2023– 15 Jul 2023	
Type of HAI (N = 138), N (%)							<0.001
CR-BSI	0 (0.0)	5 (16.7)	1 (4.8)	0 (0.0)	0 (0.0)	0 (0.0)	
VAP	11 (32.4)	15 (50.0)	13 (61.9)	4 (36.4)	16 (57.1)	8 (57.1)	
CAUTI	18 (52.9)	4 (13.3)	3 (14.3)	3 (27.3)	3 (10.7)	2 (14.3)	
BUO	5 (14.7)	6 (20.0)	4 (19.0)	4 (36.4)	9 (32.1)	4 (28.6)	
Patients with HAIs (N = 355), N (%)							0.023
No HAI	45 (68.2)	31 (58.5)	28 (62.2)	37 (80.4)	88 (82.2)	29 (76.3)	
One HAI	11 (16.7)	14 (26.4)	14 (31.1)	7 (15.2)	11 (10.3)	6 (15.8)	
Two HAIs or more	10 (15.2)	8 (15.1)	3 (6.7)	2 (4.3)	8 (7.5)	3 (7.9)	

BUO: bloodstream infection of unknown origin; CAUTI: catheter-associated urinary tract infection; CR-BSI: catheter-related bloodstream infections; HAI: healthcare-associated infection; SAPS II: simplified acute physiology score II; SD: standard deviation; SARS-CoV-2: severe acute respiratory syndrome coronavirus 2; VAP: ventilation-associated pneumonia.

**Table 3:** Multivariable competing risk Fine–Gray regression model for first HAI (N = 355).

	SHR	95% CI	*p*-Value
Period of SARS-CoV-2 variant dominance (Ref. Alpha: 1 Mar 2021–18 Jun 2021)			
Delta: 19 Jun 2021–31 Dec 2021	0.65	0.31–1.34	0.241
Omicron BA.1: 1 Jan 2022–12 Mar 2022	0.40	0.16–0.96	0.040
Omicron BA.2: 13 Mar 2022–6 Jun 2022	0.30	0.13–0.72	0.007
Omicron BA.5: 7 Jun 2022–12 Feb 2023	0.23	0.11–0.50	<0.001
Omicron XBB: 13 Feb 2023–15 Jul 2023	0.34	0.14–0.82	0.016
Age	0.99	0.98–1.01	0.463
Male gender (Ref. Female)	4.01	1.68–9.57	0.002
SAPS II	1.01	0.99–1.02	0.538
Coexisting conditions (Ref. None)			
One coexisting condition	1.24	0.74–2.08	0.414
Two coexisting conditions or more	0.98	0.57–1.70	0.949
Intubated at admission (Ref. Non-intubated)			
Intubated, other reasons	1.54	0.79–3.01	0.207
Intubated with COVID-19 pneumonia	1.82	1.08–3.07	0.025
Use of carbapenems, in days	0.87	0.82–0.93	<0.001
Use of glycopeptides, in days	0.98	0.96–1.01	0.159
Use of penicillins, in days	0.99	0.96–1.02	0.498
Time-varying coefficients			
Male gender * Time	0.92	0.88–0.97	0.002
Use of glycopeptides, in days * Time	1.00	1.00–1.01	0.001

CI: confidence interval; COVID-19: coronavirus disease 2019; HAI: healthcare-associated infection; SAPS II: simplified acute physiology score II; SHR: sub-distribution hazard ratio.

## Data Availability

The datasets used and/or analysed during the current study are available from the corresponding author on reasonable request.

## Supplementary Materials

The following supporting information can be downloaded at: https://www.mdpi.com/article/10.3390/antibiotics13090842/s1, Table S1: STROBE Statement—Checklist of items that should be included in reports of cohort studies.
